# Effect of Chicken Bone Extracts on Metabolic and Mitochondrial Functions of K562 Cell Line

**DOI:** 10.3390/ph13060114

**Published:** 2020-06-02

**Authors:** Consiglia Pacelli, Alessandro Di Cerbo, Lucia Lecce, Claudia Piccoli, Sergio Canello, Gianandrea Guidetti, Nazzareno Capitanio

**Affiliations:** 1 Department of Clinical and Experimental Medicine, University of Foggia, 71122 Foggia, Italy; consiglia.pacelli@unifg.it (C.P.); lucia.lecce@unifg.it (L.L.); claudia.piccoli@unifg.it (C.P.); nazzareno.capitanio@unifg.it (N.C.); 2 School of Biosciences and Veterinary Medicine, University of Camerino, 62024 Matelica, Italy; 3 Research and Development Department, SANY*pet* SpA, 35029 Bagnoli di Sopra, Padua, Italy; sergio.canello@forza10.com (S.C.); gianandrea.guidetti@forza10.com (G.G.)

**Keywords:** tetracyclines, bone powder extract, mitochondrial respiration, glycolysis, cell death

## Abstract

Background: Tetracyclines’ use in intensive animal farming has raised some concerns regarding the biosafety for humans. Increasing evidences have revealed the presence of these drugs in processed animal by-products, such as bone, throughout the food chain. A potential off-target of tetracyclines is the bacterial-like mitochondrial translational machinery, thereby causing proteostatic alterations in mitochondrial DNA-encoded components of the oxidative phosphorylation system. Methods: The Seahorse methodology, confocal microscopy imaging of mitochondrial potential and reactive oxygen species, and q-RT-PCR analysis of the expression of genes involved in mitochondrial biogenesis and mitophagy were carried out on human lymphoblast derived K562 cell line challenged with bone powder derived from chicken treated with or without oxytetracycline and pure oxytetracycline. Results: A complex dose-dependent profile was attained with a low dosage of bone powder extracts causing a metabolic adaptation hallmarked by stimulation of the mitochondrial respiration and enhanced expression of mitochondriogenic factors in particular in cells challenged with oxytetracycline-free bone extract. Conversely, a higher dosage of bone powder extracts, regardless of their source, caused a progressive inhibition of mitochondrial respiration and glycolysis, ultimately leading to cell death. No significant effects of the pure oxytetracycline were observed. Conclusion: Bone powder, regardless of chicken treatment, contains and releases factors/chemicals responsible for the observed effects on energy metabolism. Quantitative differential effects appear to depend on biochemical alterations in the bone matrix caused by antibiotics rather than antibiotics themselves.

## 1. Introduction

Tetracyclines are widely employed antibiotics in zootechny because of their broad spectrum of action in prophylaxis to prevent the spread of bacterial pathogen infection in farm animals [1,2,3]. Mechanistically, tetracyclines inhibit protein translation in bacteria by binding to the 30S and 50S subunit of the microbial ribosome [4]. However, tetracyclines also bind eukaryotic ribosomes accounting for the small off-site effect in mammalian cells [5]. Of note, given the similarity of bacterial ribosomes with the mitochondrial ribosome (mitoribosome), tetracyclines more specifically affect mitochondrial translation in eukaryotic cells [6].

Mitochondria have a “bacterial” ancestry and are endowed with autonomous translational machinery, distinct from that of the nucleus/cytosol, enabling biosynthesis of proteins exclusively coded by the mitochondrial DNA (mtDNA) [7]. All the mtDNA-coded proteins are subunits that along with others coded by the nuclear DNA co-assemble to form the respiratory chain complexes I, III, IV, and the ATP-synthase. All these complexes, together with complex II, constitute the terminal arm of the aerobic catabolism, coupling the transfer of reducing equivalents from NADH and FADH_2_ to O_2_ to the synthesis of ATP by a chemiosmotic mechanism [8]. As the mitochondrial oxidative phosphorylation (OxPhos) is the more efficient system to generate ATP in the cell, it is not surprising that tetracycline antibiotics influence the cell bioenergetics by affecting the mitochondrial proteostasis. This constitutes a major concern when farm animals subjected to antibiotic administration are intended for the food market. For this reason, the admitted dosage of antibiotic administration and allowed maximal residual limits are regulated by stringent guidelines [9,10]. However, tetracyclines, in particular oxytetracycline (OTC), which can accumulate in the bone tissue of farmed animals, are not included in the target samples imposed by the European Community [9] though they can be present in mechanically separated meat (e.g., sausages, Vienna sausages) [11] as well as in dry pet food [12,13,14].

In fact, according to the European Food Safety Authority’s opinion, calcium content is considered the only appropriate chemical parameter to discriminate between mechanically and nonmechanically separated meat products [11,15]. Thus, long-lasting consumption of such food might be related to some reported cases of adverse food reactions observed in humans [16,17] and pets [12,13,18]. Moreover, several in vitro reports evidenced the toxicity [14,19,20,21], proinflammatory [19,22], and genotoxic [23] activity of bone powder derived from chicken treated with OTC according to withdrawal times but also of OTC in its liquid form as per generally used in zootechny.

On this basis, this study aimed to verify the effect of extracts from bone powder derived from chicken treated with or without OTC according to withdrawal times [20] on the viability and metabolic profile of the cultured human lymphoblast derived K562 cell line with a specific focus on the mitochondrial functions.

## 2. Results

### 2.1. Effect of Chicken Bone Powder Extracts and Oxytetracycline on K562 Cell Viability

Samples of chicken bone powder were obtained from broiler chicken raised either under natural/biological and tetracycline-treated conditions. The OTC content in chicken bone extracts was evaluated by LC-MS/MS spectrometry, and the results were below the instrumental limit of detection (i.e., < 0.5 μg/kg) in untreated chicken and 1286.3 ± 256.6 μg/kg) in drug-treated chicken [20].

**Figure 1:**
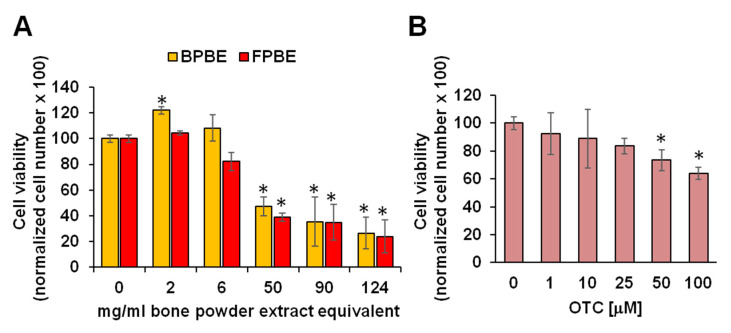
Effect of bone powder extracts on K562 cell viability. Cells were incubated for 24 h with the indicated concentrations of either biologically raised poultry bone extract (BPBE) and intensive-farming raised poultry bone extract (FPBE) (**A**) or oxytetracycline (OTC) (**B**) and directly counted by the Trypan Blue exclusion test. Bars are means ± SEM of four independent biological replicates for each condition and are percentages of vehicle-treated cells; * *p* < 0.05 vs. 0 g/mL of bone extracts or 0 mM OTC.

The K562 cell line has attained widespread use as a highly sensitive in vitro target for testing cytotoxicity of a variety of compounds [24,25]. Figure 1A shows the effect on cell viability of 24 h treatment of K562 cells with graded dilutions of RPMI extracts from bone powder derived from poultry raised on the ground in a full natural context, from here ahead defined as BPBE (biologically raised poultry bone extract). It can be seen that cell viability was unaffected at relative low dilutions of BPBE corresponding to 2 and 6 mg/mL of bone powder but started to decrease progressively with BPBE from 50 to 124 mg/mL of bone powder with 50% reduction of viability at the lowest dilution tested. A similar test was performed on K562 cells, but following incubation with extracts from bone powder derived from poultry raised in farm, from here ahead defined as FPBE (intensive-farming raised poultry bone extract). The results are illustrated in Figure 1B and show that similar to BPBE, FBPE caused a dose-dependent decrease of cell viability through starting at a dilution corresponding to 6 mg/mL of the bone powder. For comparative purposes, K562 cells were incubated with pure OTC in the concentration range of 1–100 μM and Figure 1B shows that OTC affected cell viability significantly at 50 and 100 μM.

### 2.2. Effect of Chicken Bone Powder Extracts and Oxytetracycline on Mitochondrial Respiration and Glycolysis in K562 Cell

**Figure 2:**
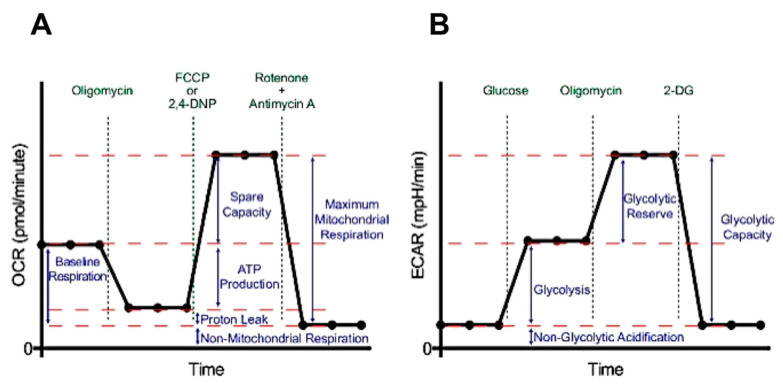
Representative outcome of the metabolic flux analysis protocol. (**A**) Oxygen consumption rate (OCR) was first measured under routine condition (baseline respiration), and then its changes following the sequential additions of oligomycin, carbonyl cyanide p-trifluoromethoxyphenylhydrazone (FCCP), rotenone + antimycin A; the bioenergetic parameters inferred from the comparison of the different OCR attained are shown. (**B**) Extracellular acidification rate (ECAR) was first measured in a buffer without glucose, and then its changes following the sequential addition of glucose, oligomycin, and 2-deoxyglucose (2-DG); the glycolytic parameters inferred from the comparison of the different ECAR attained are shown.

To correlate the observed effect of BPBE, FBPE, and OTC on cell viability to alterations of the cellular metabolism, we performed a systematic analysis using the Seahorse technology to assess the two major cellular metabolic fluxes [26]. These are the mitochondrial respiratory activities driving the oxidative phosphorylation (OxPhos) and measured as oxygen consumption rate (OCR), and glycolysis, measured as extracellular acidification rate (ECAR), largely due to conversion of pyruvate to lactate. Figure 2A,B shows the workflow and outcomes of this test.

For the mitochondrial respiratory activity after measuring the basal OCR (OCR_bas_), the inhibitor of the ATP synthase oligomycin was added to elicit a lower membrane potential-controlled OCR (OCR_olig_); after that, the protonophore uncoupler Carbonyl cyanide 4-(trifluoromethoxy)phenylhydrazone (FCCP) was added to release the maximal respiratory capacity (OCR_unc_) and finally a combination of the two respiratory chain inhibitors antimycin A + rotenone to set a reference baseline and to assess nonmitochondrial OCR. For measurement of glycolysis, cells in glucose-free medium were supplemented with glucose and basal ECAR assessed (ECAR_bas_); after that, oligomycin was added to elicit the maximal glycolytic capacity (ECAR_max_) and finally the glycolysis inhibitor 2-DG to set a reference baseline and to assess nonglycolytic ECAR.

**Figure 3:**
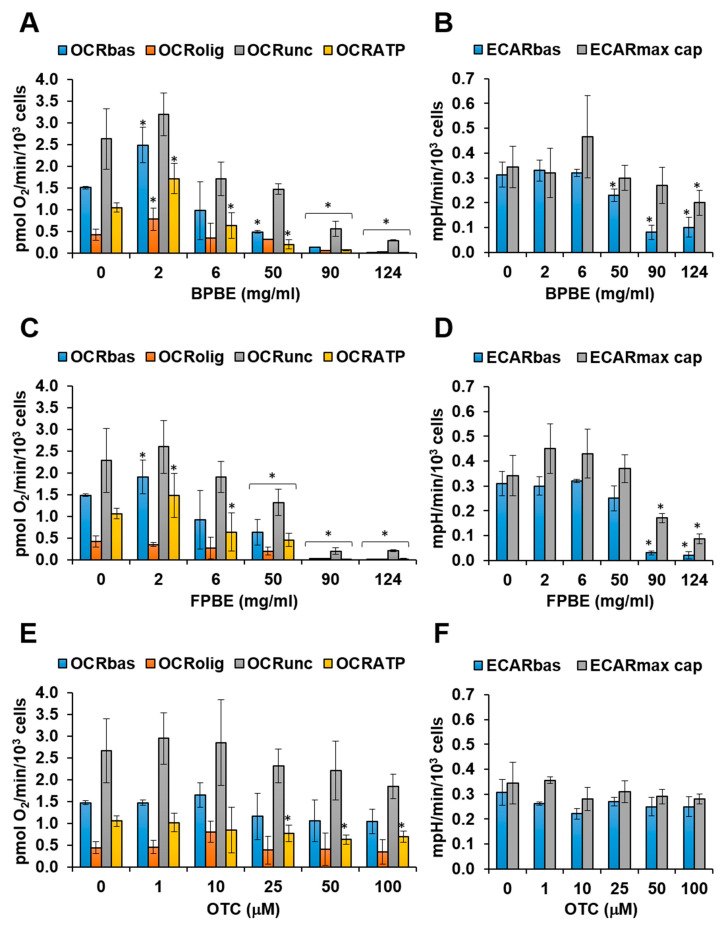
Effect of chicken bone powder treatment on the metabolic fluxes in K562 cells. Cells were incubated for 48 h with the indicated concentrations of either BPBE (**A**,**B**) and FPBE (**C**,**D**), or OTC (**E**,**F**) and then subjected to metabolic flux analysis as detailed under Materials and Methods. The different OCRs and ECARs parameters are detailed in Figure 2. Bars are means ± SEM of 3–4 independent experiments with three technical replicates each; * *p* < 0.05 with respect to the same parameter in untreated cells (“0” concentration of the compound).

Using the above-described protocol, the effect of BPBE on the metabolic fluxes of K562 was tested. As shown in Figure 3A, BPBE treatment resulted in a biphasic effect on the mitochondrial respiratory activity; at the highest dilution of the BPBE corresponding to 2 mg/mL of bone powder, the OCR_bas_ almost doubled as compared with untreated cells but started to decline progressively at lower dilution of BPBE to an extent that at the lowest dilution tested the OCR_bas_ was barely detectable. In keeping with the notion that the difference between the OCR_bas_ and the OCR_olig_ is an indirect measure of the mitochondrial respiration linked to ATP production (OCR_ATP_), it is worth noting that it followed the same profile of the OCR_bas_, thereby providing a measure of the OxPhos efficiency. Unlike OCR, glycolysis appeared to be less sensitive to BPBE. Indeed, ECARs, though inhibited at the lower dilution of BPBE, still retained a 40%–50% of residual activity as compared with untreated cells (Figure 3B).

The effect of FPBE on the metabolic fluxes of K652 recapitulated what was observed with BPBE with some differences. In particular, the stimulation of the mitochondrial respiration at the highest dilution of FPBE was smaller than that attained with BPBE, and the inhibition of ECAR_bas_ was stronger at the lowest dilutions of FPBE (Figure 3C,D). Pure OTC treatments did not result in any significantly large changes in the respiratory activity, with the exception of an increase at 10 μM, and in a progressive decline of the glycolytic activity which, however, retained about 50% of residual ECARs at the highest concentration of OTC tested (Figure 3E,F).

### 2.3. Effect of Chicken Bone Powder Extracts and Oxytetracycline on the Mitochondrial Membrane Potential and on Peroxide Production in K562 Cell

**Figure 4:**
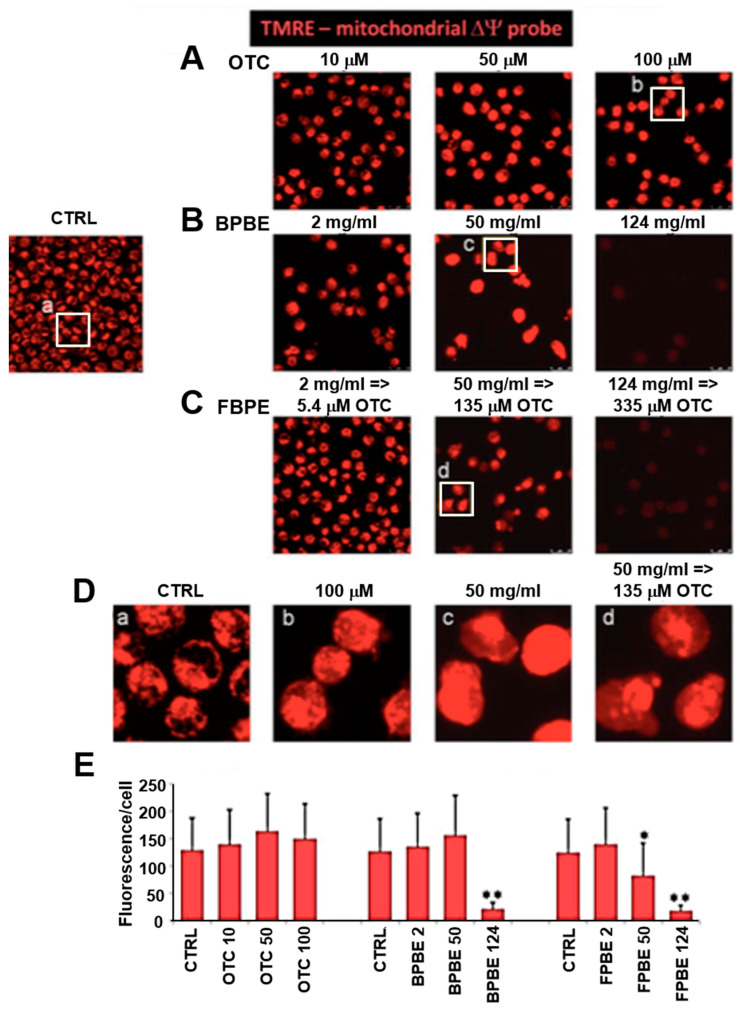
Effect of chicken bone powder treatment on the mitochondrial membrane potential in K562 cells. Cells were treated for 48 h with the indicated concentrations of either of OTC (**A**), BPBE (**B**), FPBE (**C**), and then incubated with tetramethylrhodamine, ethyl ester (TMRE) and subjected to confocal microscopy analysis as detailed under Materials and Methods; untreated cells were used as control (CTRL). The images shown are from a single experiment and are representative of three independent experiments yielding similar results; (**D**) digital magnification of selected areas shown as white frames. (**E**) Quantification of the fluorescence-related pixel intensity/cell; for each condition and for each experiment 10–15 different optical fields were randomly chosen, and the fluorescence intensity/cell averaged ± SEM; * *p* < 0.05, ** *p* < 0.01 with respect CTRL cells.

To provide independent evidences of the effect of the compound treatments on the mitochondrial activity, we utilized laser scanning confocal microscopy to image the respiration-driven mitochondrial membrane potential (ΔΨm) in live cells using the mitotropic fluorescent probe tetramethylrhodamine, ethyl ester (TMRE) [27]. For this analysis, we selected only three conditions corresponding to low, intermediate, and high concentrations of the compound’s treatment. The results illustrated in Figure 4B,C show that at the lowest dilution tested, both BPBE and FPBE treatment caused an increasing reduction in ΔΨm consistent with the inhibition of the mitochondrial respiratory activity. No significant changes in the intensity of the ΔΨm-related fluorescent signal was detectable at low and intermediate concentrations. On the contrary, pure OTC treatment of K562 cells did not result in appreciable changes of the TMRE fluorescence at all the concentrations tested, Figure 4A. Enlargements of the images allow the appreciation of the intracellular distribution of the fluorescent signal (Figure 4D); in the untreated control cells, the signal was localized in an interconnected extranuclear compartment clearly resembling the mitochondrial network and a comparable distribution was observed in cells treated with 100 μM OTC.

Conversely, in cells treated with intermediate dilutions of both BPBE and FBPE, the TMRE-related fluorescent signal appeared diffused and/or concentrated in aggregate indicative of an unstructured mitochondrial compartment, thereby anticipating the metabolic sufferance observed following treatment of the cells at the lowest dilutions of the bone extracts.

**Figure 5:**
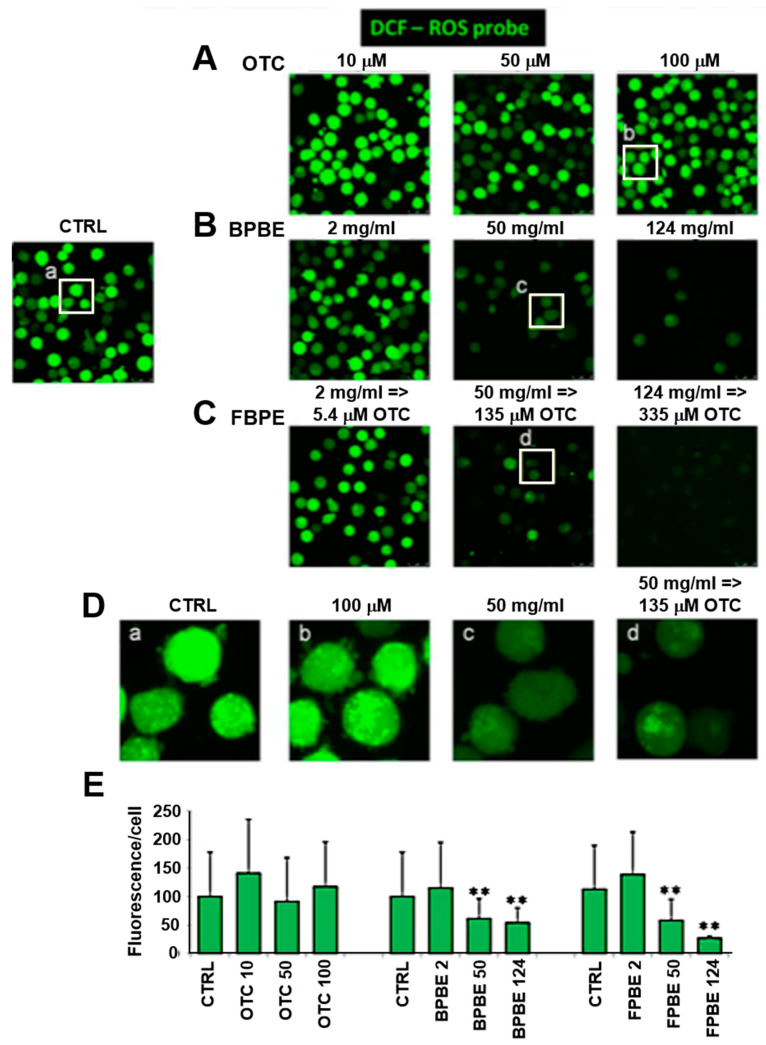
Effect of chicken bone powder treatment on the reactive oxygen species production in K562 cells. Cells were treated for 48 h with the indicated concentrations of either of OTC (**A**), BPBE (**B**), FPBE (**C**), and then incubated with 2’,7’-dichlorodihydrofluorescein diacetate (H_2_-DCFDA) and subjected to confocal microscopy analysis as detailed under Materials and Methods; untreated cells were used as control (CTRL). The images shown are from a single experiment and are representative of three independent experiments yielding similar results; (**D**) digital magnification of selected areas shown as white frames. (**E**) Quantification of the fluorescence-related pixel intensity/cell; for each condition and for each experiment, 10–15 different optical fields were randomly chosen, and the fluorescence intensity/cell averaged ± SEM; ** *p* < 0.01 with respect CTRL cells.

The mitochondrial respiratory chain is the major source of reactive oxygen species (ROS) in the cell which are generated by electron leak directly to oxygen with the production of the superoxide radical anion O_2_^•-^ further converted to H_2_O_2_, by the mitochondrial superoxide dismutase, and in this form diffuses outside the organelle [28,29]. On this basis, we monitored the production of cellular ROS as an indirect measure of the respiratory activity by the peroxide sensitive fluorescent probe dichlorofluorescein (DCF) [30]. As shown in Figure 5, both BPBE and FPBE treatment caused a significant reduction in the DCF-related signal at the intermediate and lowest dilutions. Conversely, no changes were observed at all the concentrations of pure OTC tested.

### 2.4. Effect of Chicken Bone Powder Extracts and Oxytetracycline on the Expression Level of Factors Involved in the Mitochondrial Turn-Over

Mitochondria are dynamic organelles undergoing continuous cycling as a consequence of the balance between their biogenesis and mitophagic removal, thereby adapting their relative content to the actual cellular energy demand [31]. To investigate this aspect of the mitochondrial physiology, we analyzed the expression level of selected genes coding for markers of mitochondria and for transcription factors known to be involved either in mitochondrial biogenesis and mitophagy by quantitative RT-PCR. We could not extend this analysis to K562 cells treated with the lowest dilutions of both BPBE and FPBE because the limited amount of total cellular RNA extract hampered the acquisition of reproducible results. Of note, however, at the lowest and intermediate dosage of the extracts tested, the amount of recovered RNA was about 50% lower than that attained by untreated cells (data not shown).

**Figure 6:**
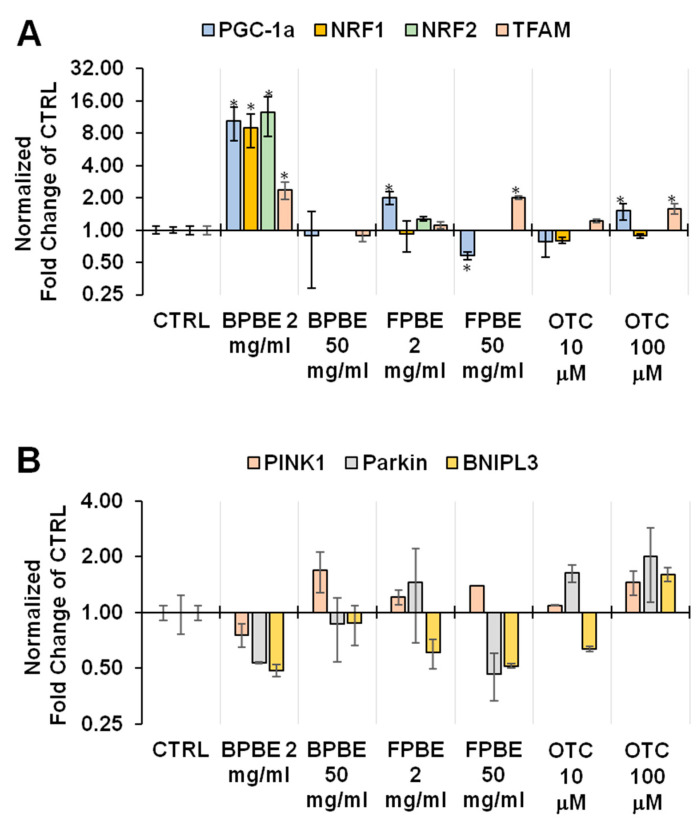
Effect of chicken bone powder treatment on the transcription of selected genes involved in mitochondriogenesis and mitophagy in K562 cells. Cells were treated with either BPBE, FBPE, or OTC at the indicated concentrations and subjected to quantitative RT-PCR on extracted RNA as detailed in Materials and Methods. The expression of the indicated selected genes involved in the mitochondrial biogenesis (**A**) or mitophagic processing (**B**) is shown as fold changes of the untreated (CTRL) cells (in a log scale); bars are means ± SEM of three independent experiments with three technical replicates under each condition; *p < 0.05 as compared with CTRL.

Figure 6A shows the normalized transcript levels of the genes coding for the peroxisome proliferator-activated receptor-gamma coactivator 1-alpha (PGC-1α), a master regulator of the mitochondrial biogenesis and for the mitochondrial transcription factor A (TFAM), a key activator of the mitochondrial genome transcription and replication [32]. Notably, low concentrations of BPBE caused a statistically significant ten-fold increase in the transcription level of PGC-1α, whereas FPBE at the same dilution elicited a smaller two-fold increase of the same transcript although still statistically significant. To support the effect on the PGC-1α transcription observed at low concentrations of BPBE, we also checked the transcript levels of two genes coding for the nuclear transcription factors 1 and 2 (NRF1, NRF2), which are also involved in mitochondrial biogenesis but, in turn, are controlled by PGC-1α [32]. The results obtained show that both NRF1 and NRF2 expression was about ten-fold higher following low-dosage BPBE-treatment as compared with untreated cells. A two-fold increase in NRF2 was also observed at low-dosage FPBE-treatment. At intermediate dilution of both the bone extracts, a sharp decline of the PGC-1α transcript level was observed. Conversely, no significant changes were observed following treatment with the pure OTC at the two concentrations tested. Finally, we measured the expression level of genes coding for factors involved in the mitochondrial quality control, which is a multi-steps process whereby cells activate a selective removal of damaged mitochondria in response to stressing conditions [33]. PINK1, Parkin, and BNIP3L (also known as NIX) are proteins that target mitochondria for degradation into intracellular autophagosomes. Analysis of the transcription levels of these mitophagic factors revealed a complex compound- and dose-dependent profile with a significant 50% reduction in the transcript level of all the three factors only under the condition of low-dosage BPBE-treatment (Figure 6B).

## 3. Materials and Methods

### 3.1. Chicken Bone Extract

The treatment of chicken was in accordance with the guidelines of European and Italian laws, and the study protocol was as detailed in [20]. Briefly, thirty-six 1-day-old male and female broiler chickens (Ross 708) were randomly assigned to 2 groups, the control (n = 18) and treated (OTC) animals (n = 18). The oxytetracycline-treated group received OTC in its liquid form (Ossitetraciclina liquida 20%, TreI, Reggio Emilia, Italy) at a dosage of 40 mg/kg live weight via drinking water at day 1, 5, 20, and 25. The withdrawal time, at day 10, was applied between the last OTC administration and slaughtering, that occurred on day 35. After slaughtering, the bones collected from deboned breast and leg were dried, broken, autoclaved, finely ground, and stored at −20 °C. For the evaluation of OTC, ground bone samples were treated with McIlvaine-Na_2_ EDTA 0.1 M buffer (pH 4) and the extracts assayed by liquid chromatography-electrospray interface (ESI) mass spectrometry (MS)/MS system (Walnut, Creek, CA, United States). The instrument was calibrated with a known amount of OTC, and the limit of detection was 0.5 mg/kg (see [20] for further details).

### 3.2. Cell Culture and Compound Treatment

The human lymphoblast derived K562 cell line was purchased from the American Type Culture Collection (ATCC, Manassas, Virginia, USA) and cultured at 37 °C in a humidified atmosphere (5% CO_2_) in RPMI (Sigma–Aldrich, St. Louis, Missouri, USA) medium for 48 h. Oxytetracycline (OTC) from Sigma–Aldrich (St. Louis, Missouri, USA) was stocked at 100 mM in DMSO (Sigma–Aldrich, St. Louis, Missouri, USA). Bone powder, achieved from poultry raised with or without the administration of oxytetracycline, was extracted in cell culture medium as previously described [19,20]. Briefly, the bone powder was dissolved in RPMI at the concentration of 124 mg/mL and kept under continuous stirring at 37 °C for 48 h. After that, the suspension was filtered, and the filtrate neutralized with KOH (Sigma–Aldrich, St. Louis, Missouri, USA) at pH 7.2–7.4. For compound treatment, 0.5–2.0 × 10^6^ cells/mL were supplemented with graded concentration of OTC or graded dilution of bone powder extracts as indicated. The viability of K562 cells was assessed before and after treatments by direct counting Trypan Blue-negative cells with a hemocytometer.

### 3.3. Metabolic Flux Analysis

Oxygen consumption rate (OCR) and extracellular acidification rate (ECAR) were measured with an XF96 Extracellular Flux Analyzer (Seahorse Bioscience, Billerica, MA, USA) in RPMI following cytospinning of the K562 cells in the wells of the Seahorse cartridge. The medium was supplemented with 1 mM pyruvate, 2 mM glutamine, 10 mM glucose for OCR measurements or with 1 mM pyruvate, 2 mM glutamine for ECAR measurements. Briefly, for OCR analysis, after measuring basal respiration, oligomycin (1 μM), FCCP (1 μM), and rotenone + antimycin A (1 μM + 1 μM) were injected into each well sequentially to assess coupling of the respiratory chain, maximal and nonmitochondrial oxygen consumption, respectively. For ECAR analysis, glycolytic flux (basal glycolysis, glycolytic capacity, and glycolytic reserve) was analyzed by the sequential addition of 10 mM glucose, 1 μM oligomycin, and 100 mM 2-deoxyglucose. OCR and ECAR values were normalized to the cell number in each well.

### 3.4. Confocal Microscopy Imaging

Cells were cytospun on fibronectin-coated (Cell-tak-coated (Corning, New York, USA)) 35-mm glass-bottom dishes (Ibidi, Gräfelfing, Germany)] and incubated for 20 min at 37 °C with 100 nM Tetramethylrhodamine, ethyl ester (TMRE, Life Technologies, Carlsbad, CA, USA) to detect the mitochondrial membrane potential or with 5 μM DCF-DA (2,7-dichlorofluorescin diacetate) (Molecular Probes, Eugene, Oregon, USA) to detect cellular peroxide. Stained cells were washed with PBS and examined by a Leica (Wetzlar, Germany) TCS SP8 confocal laser scanning microscopy system utilizing appropriate excitation laser beams (images collected using a 60X objective (1.4 NA)). Acquisition, storage, and analysis of data were performed with LasX software from Leica or ImageJ 1.48 (Wayne Rasband, NIH, Bethesda, Maryland, USA).

### 3.5. RNA Isolation and Quantitative RT-PCR

Total RNA from the K562 cell line was extracted using an Aurum Total RNAMini Kit (Bio-Rad, Hercules, CA, USA) according to the manufacturer’s protocol. 0.5 mg of total RNA was then reverse-transcribed to generate cDNA for PCR by using the iScript cDNA Synthesis kit (Bio-Rad, Hercules, CA, USA). Semi-quantitative determination of mRNA levels was performed by real-time qRT-PCR using SYBR Green (Bio-Rad, Hercules, CA, USA). Reactions were performed in duplicate for each sample. Multiple reactions were performed in a volume of 20 μL containing 10 μL of 2 × PCR master mix, 1X of validated specific primers (Qiagen, Hilden, Germany) (see Table 1), and 2 μL of cDNA template. Amplifications were performed in the Stratagene MX3000P Real-Time PCR Detection System (Stratagene, San Diego, CA, USA), using the following cycle program: denaturation step at 95 °C for 10 min followed by 40 cycles of denaturation at 95 °C for 15 s, annealing at 55 °C for 1 min, and extension at 72 °C for 30 s. The relative mRNA expression levels were calculated using the comparative CT method (2^-ΔΔCT^). Quantitative normalization for each sample was performed by using glyceraldehyde-3-phosphate dehydrogenase (GAPDH, Qiagen, Hilden, Germany) as an internal control.

### 3.6. Statistical Analysis

All the experiments were carried out in triplicate. Data are shown as mean ± standard error mean (SEM) and compared by the unpaired Student’s *t*-test or one-way ANOVA, followed by Bonferroni post-hoc test; a *p-value < 0.05 was accepted as statistically significant. All analyses were performed using Graph Pad Prism (Graph Pad software, San Diego, CA, USA).

## 4. Discussion

**Figure 7:**
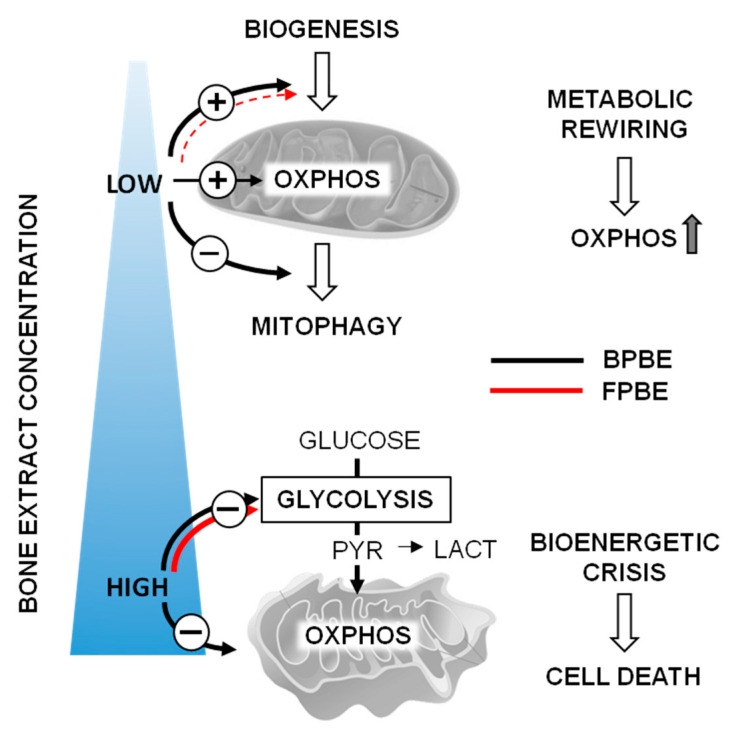
Schematic drawing of the major effects of chicken bone extracts on mitochondrial function described in this study; the circled “+” an “−“ indicate stimulation and inhibition, respectively, of the indicated function with the thickness of the line proportional to the intensity of the effect. OXPHOS, oxidative phosphorylation; PYR, pyruvate; LACT, lactate. See Discussion for further details.

The results presented in this study allow drawing the following conclusions schematized in Figure 7.

The bone extracts from chicken raised with or without prophylactic antibiotic administration exert complex dose-dependent effects on the metabolism of the K562 cells. The outcome profile appeared to be biphasic, being cytotoxic at relatively high concentrations but metabolism-stimulating at very low concentrations. The metabolic stimulation consisted essentially of an increased mitochondrial respiration with limited effects on glycolysis. Consequently, this translated into a metabolic shift toward a more efficient ATP-generating mitochondrial OxPhos. Comparatively, the BPBE appeared slightly more efficient than FPBE to elicit the low-dosage-mediated metabolic shift. Assuming that the amount of OTC extractable from FPBE is in the order of 1.2–1.3 μg of the antibiotic/Kg bone powder [20], it is easy to calculate that in our condition the K562 cells treated with FPBE were likely exposed to a concentration of OTC ranging from 5.4 to 335 μM at the highest and lowest dilution of the extract, respectively. On this basis, as the pure OTC at concentrations comparable with that released in the FPBE at the highest dilution did not cause appreciable metabolic changes, this would rule out a contribution of the antibiotic to the differential metabolic impact observed in the BPBE- and FPBE-treated cells. Higher concentrations of bone extracts, regardless of their source, caused a progressive inhibition of both the mitochondrial respiratory activity and glycolysis, though more pronounced in the former. This established a condition of bioenergetic crisis with a consequent reduction in cell viability.

Mechanistically, the observed low-dosage-mediated stimulation of the aerobic metabolism appeared to be linked to changes in the expression of genes controlling the mitochondrial biogenesis and/or the mitophagic removal of the organelles. A combination of these two processes was particularly evident at the lowest concentration of BPBE and FPBE, though to a much lesser extent in the latter. In addition, in this case, the differential transcriptional impact of FBPE treatment cannot be ascribed to antibiotics since pure OTC did not result in significant changes in the transcription profile of the analyzed factors. At higher concentrations of the bone extracts, the stimulatory effect on the mitochondriogenic transcription factors was lost, and other effects prevailed leading to cell sufferance and death.

## 5. Study Limitations

The major limitation of this study is that it was carried out on a single cell line. However, in a previous investigation, we challenged human peripheral blood mononuclear cells (PBMC) with bone extracts observing a proinflammatory effect [20]. Extension of this study on the specific effect of the bone extract on the mitochondrial function of different primary cells and cell lines is ongoing in our labs. A further limitation is that our findings do not enable us to draw definitive conclusions on their impact on the consumer. The FDA and WHO have recently established residue limits of tetracyclines in several alimentary matrices (such as liver, kidney, fat) but not for bone that is considered inedible, in spite of the fact that tetracyclines have a high affinity for calcium-rich tissues, such as bone and teeth. The effect, here shown, exerted by chicken bone extracts, even at very low concentrations, in modifying the cellular energy metabolism is a warning for the need for further in-depth investigations to define the effect of alimentary bone-derivative supplements on the consumer health.

## 6. Conclusions

In conclusion the presented results suggest that under the experimental conditions tested in this study, bone powder contains and releases factors/chemicals, that regardless if derived from naturally (antibiotic-untreated) or intensively farmed (antibiotics-treated) poultry, are responsible for the observed effects on mitochondrial metabolism. The quantitative differential effects observed between BPBE- and FPBE-treatment appear to depend on biochemical alterations in the bone matrix caused by antibiotics rather than by the antibiotics themselves [1,2]. In addition to the identification of the chemical nature of the bioactive compounds released from the bone matrix, it remains to be established (i) if other cells, phenotypically different from the K562 cells tested in this study, rewire their metabolism following low-dosage bone extract-treatment and (ii) the mechanism(s) of cell death occurring at high-dosage treatment.

## Figures and Tables

**Table 1:** List of primers used to amplify the transcripts of the indicated genes by q-RT-PCR.

Gene	Product Number
*PPARGC1A*	QT00095578
*TFAM*	QT00012782
*NRF1*	QT01154076
*NRF2*	QT01192688
*PINK1*	QT00056630
*PARK2*	QT00023401
*BNIP3L*	QT00022939
*GAPDH*	QT00079247
